# Supplementary material for: The effect of a digital targeted client communication intervention on pregnant women’s worries and satisfaction with antenatal care in Palestine–A cluster randomized controlled trial
Source: PLoS One. 2021 Apr 23;16(4):e0249713. doi: 10.1371/journal.pone.0249713 (PMC8064599; doi:10.1371/journal.pone.0249713)
Supplement: Attachment — Submitted filename: Response to Reviewers.docx [file pone.0249713.s003.docx]

**Reviewer #1:**

It is a nicely written manuscript. Just a few comments. The outcome of the study is presented as "improved attendance and quality of care" yet the AIM of the sub-study is stated - to assess unintended consequences of the TCC intervention on pregnant women's worries, and their satisfaction with antenatal care.

Our response:

Thank you for identifying this. The statement was referring to the primary outcomes of the effectiveness trial on which this sub-study is based. This paper presents an additional data collection and analysis with the aim of assessing any unintended consequences of the intervention. We assessed whether women’s worries during pregnancy, and their satisfaction with the antenatal care service, could be negatively affected by the intervention.

We have clarified this in statements in line 25 and 84.

In Ln 75 the statement is made that the authors have developed a theory based, co-designed and used and tested TCC, but it sounds as if this was a secondary analysis of existing data. If so, this needs to be clarified....or consider rephrasing along the lines of "in this secondary analysis, data using the theory-based ...."

Our response:

This relates to the same unclarity as above. This paper represents a new data collection to assess any unintended consequences of a trial, which had the primary objective of improving attendance and quality of care. To clarify our point, we added sentence with reference to previous study in line 74-75, and rephrased the sentence in line 84.

The abstract states that the primary reason for the TCC was to improve the quality of care yet the methods section goes into a lot of information about increasing awareness, improving attendance...were all these components assessed? Please align the abstract with the paper.

Our response:

Thank you for pointing out this for clarification. We have revised the abstract accordingly. Lines 25 and 137

Be careful to use 'past-tense' terminology throughout the paper, Ln 106, 'were' applied, not 'are' applied, Ln 108, 'were' applied not 'are' applied.
Ln 138, Difference of 'each question' not 'each questions.'
Ln 245 - Validate instead of 'validating'

Our response: thank you or identifying the errors. We corrected them.

Nice statement about taking precautions in composing individualized messages and giving special attention to messaging for pregnant women.

Thank you. This was our rationale in conducting this sub-study.

Reviewer #2: A non-inferiority designed substudy was conducted to assess targeted client communication (TCC) intervention to no TCC (control) on pregnant women’s worries and satisfaction with antenatal care.

Minor revisions:
1- Line 156: Typographical error: Model.

Our response:

Thank you for identifying the error. Corrected.
2- Line 156: State the underlying covariance structure used in the mixed effect model and the criteria for selecting it.

Our response:

Thank you for your suggestion. Now we have added more text into the methods section, line 162-164

3- Table 1: Include p-values to compare background characteristics between the intervention and control arms. Additionally, in the statistical method section, indicate the statistical methods used to estimate the p-values.

Our response:

Thank you for raising this point. We believe we will leave this for the Editors to decide as a matter of journal policy. We have followed the CONSORT statement that recommends not to report significance tests of baseline characteristics (“Standard errors and confidence intervals [and therefore P-values] are not appropriate)”. If the Editors prefer the inclusion of significance tests, we will be happy to provide them.

4- Tables 2 and 3: Provide an interpretation for each level of worry and level of agreement. Would these levels be considered a likert scale? Please clarify.

Our response:

Yes, both for worries and satisfaction, these are Likert-type scales where the respondents rate their agreement/disagreement with statements. We have added interpretation of the levels in the footnotes under Table 2 and 3.

Reviewer #3: Dear Authors
1. The problem for intervention is not clear. I could not understand what is the problem of Palestinian women in antenatal care?

Our response:

Thank you for the question. We have now added a sentence to indicate the gap our intervention intended to fill in lines 74-75, referring to a previous assessment of attendance and quality of care in Palestine.

2. There is no relation between intervention with outcome assessment tool. The Authors conducted a digital targeted client communication intervention with SMS to attended for receiving prenatal care. After intervention they assessed the worries?

Our response:

Please see responses to reviewer #1.

What was the content of message to reduction of worries?

Our response:

Our TCC intervention was aimed at improving attendance, not specifically reducing worries. Increased worries would be an unintended consequence of messaging to increase attendance. As we have reported in detail elsewhere, this intervention included messages that focused on potential risks in pregnancy, and how attendance to ANC could reduce risks. We wanted to study whether this kind of messages could affect worries in pregnancy.

3. The authors concluded that "The TCC intervention via SMS did not increase pregnancy related worries among recipients"
I think that the authors did not select a suitable assessment tool for the intervention.

Our response:

Please refer to comments above. The assessment tool, the Cambridge Worry Scale has been validated in multiple settings.

4. The inclusion and exclusion criteria is not written well.

Our response:

For the overall trial criteria, we have included the reference to the protocol paper where detailed eligibility criteria were presented. For the specific inclusion and exclusion criteria for the additional data collection of this sub-study, we have clarified in lines 104-106

5. The blindness and masking?

Our response:

Thank you for raising this question. For the data collection and analysis, we have stated that the data collectors were blinded for allocation and primary outcome (line 128), and the statistician was blinded for allocation (line 169). Otherwise, the nature of the intervention does not allow for blinding and masking participants.

6. Fig participants is not clear. At initiation of study four arms and then two arm? Why?
I overall, I was confused. I cloud not find a suitable deification of problems, the intervention,, and assessment tool. I do not understand that why the main results of the trial is factors of worries of pregnant women

Our response:

Please refer back to previous responses. How the participant flow chart came about is additionally explained in lines 95-106.
