# Supplementary material for: The effect of a digital targeted client communication intervention on pregnant women’s worries and satisfaction with antenatal care in Palestine–A cluster randomized controlled trial
Source: PLoS One. 2021 Apr 23;16(4):e0249713. doi: 10.1371/journal.pone.0249713 (PMC8064599; doi:10.1371/journal.pone.0249713)
Supplement: Attachment — Submitted filename: Response to the academic editor.docx [file pone.0249713.s004.docx]

Response to the academic editor

Thank you for the comments and edits. We accepted all your comments and recommendations for edits.

Regarding your question about validation of CWS in Arabic language, we added a phrase “(has not been validated in Arabic language)” in Line #250.

Best regards,
